# Segmental late gadolinium enhancement and gadolinium extracellular volume in hypertrophic cardiomyopathy

**DOI:** 10.1186/1532-429X-18-S1-P155

**Published:** 2016-01-27

**Authors:** Jonathan M Levine, Jeremy D Collins, Gillian Murtagh, Michael Markl, James C Carr, Lubna Choudhury

**Affiliations:** Northwestern University Feinberg School of Medicine, Chicago, IL USA

## Background

Late gadolinium enhancement (LGE) by cardiac magnetic resonance (CMR) is a common finding in patients with hypertrophic cardiomyopathy (HCM), and its extent correlates with clinical parameters such as ventricular tachycardia and sudden cardiac death. While LGE has been shown to reflect focal replacement fibrosis, recently optimized T1 mapping techniques have allowed for the quantification of extracellular volume (ECV), a measure of the diffuse interstitial fibrosis also observed in HCM. However, the relationship between LGE and ECV in HCM is not fully understood. The aim of this study was to evaluate the relationship between LGE and ECV in patients with HCM.

## Methods

A retrospective chart review identified patients with a diagnosis of HCM referred for CMR at Northwestern Memorial Hospital between 1/1/2012 and 12/31/2014. Conventional segmented, inversion-recovery gradient recalled echo imaging was used to generate a stack of short axis images for LGE quantification using automated software (Medis). T1 mapping utilized pre- and 12-25 minutes post-contrast balanced steady state free precession (bSSFP) single-shot Modified Look-Locker inversion-recovery with data acquisition over 11 heartbeats. Three short axis slices (apical, mid-chamber, basal) were used to calculate ECV with hematocrit values from blood samples. Patients received 0.2 mmol/kg gadopentetate dimeglumine or 0.1 mmol/kg gadopentetate dimeglumine contrast at CMR. Segmental LGE and ECV values were calculated according to the AHA 16 segment model and compared. To characterize the presence of LGE in segments with varying degrees of ECV, patients were separated into LGE(+) and LGE(-) groups based on the presence of visually identifiable scar. All segments from LGE(+) patients were defined as LGE(+) and those from LGE(-) patients as LGE(-). All segments were pooled and grouped by range of ECV, and the total number of LGE(+) and LGE(-) segments for each ECV range was calculated.

## Results

**Figure 1:**
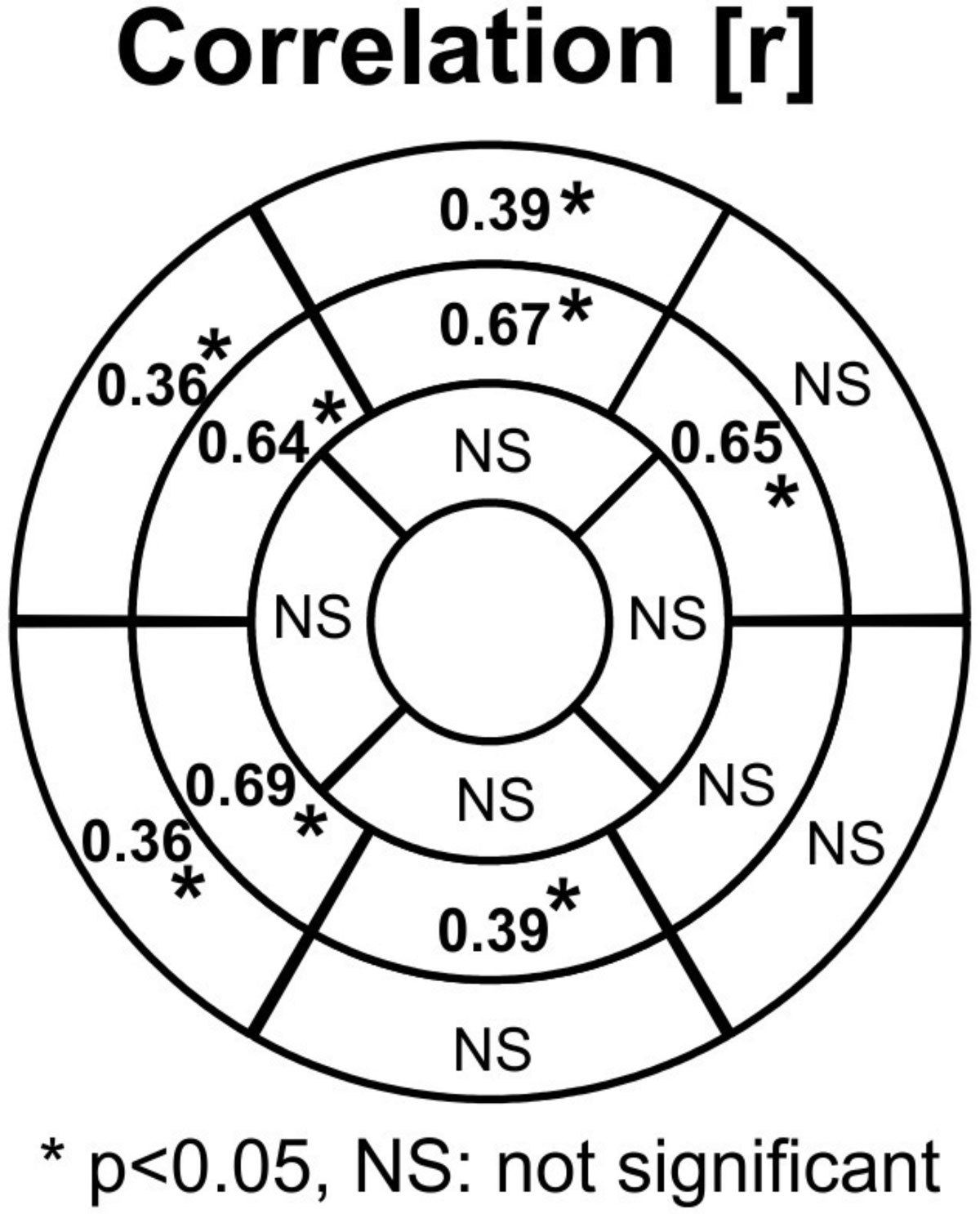
**Correlation between LGE and ECV by myocardial segment**.

**Figure 2:**
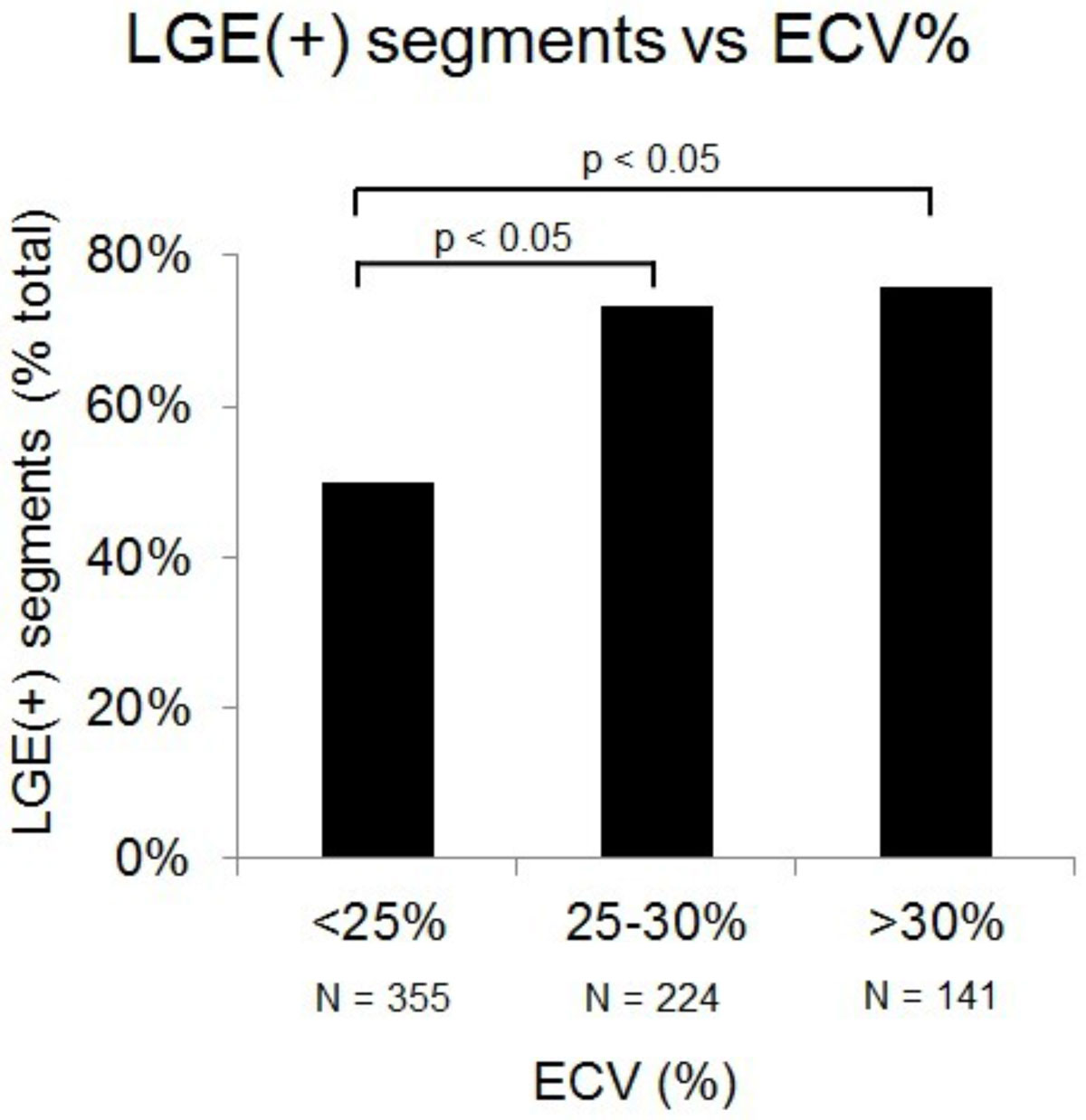
**Presence of LGE in myocardial segments with varying levels of ECV**.

45 patients (23 men, mean age 53.4 ± 13.5 years) met criteria, allowing for the evaluation of 720 myocardial segments. 37.8% of patients (n = 17) had no visually identifiable scar. Segmental LGE ranged from 0-99.7% (mean 14.5 ± 17.5%) and segmental ECV ranged from 5.65-64.2% (mean 26.2 ± 6.71%). There was a statistically significant correlation (p < 0.05) between ECV and LGE in 8 of 16 myocardial regions (Figure 1). LGE was present in 49.9%, 73.2%, and 75.9% of segments with ECV < 25% (n = 355), 25-30% (n = 224), and > 30% (n = 141) respectively (Figure 2). ECV > 30% was found in 12.5% of LGE(-) segments.

## Conclusions

The relationship between LGE and ECV in HCM varies by myocardial region, with mid-septal and antero-septal segments demonstrating the strongest correlations. Segments with greater ECV expansion demonstrated a significantly greater presence of LGE, but significant interstitial fibrosis can also occur in HCM patients with no visible scar. Further study is indicated for evaluating the significance and independent prognostic value of ECV in HCM.

